# Clinical therapeutic effects of acupuncture in treating patients with dysphagia after radiotherapy in nasopharyngeal carcinoma: A protocol for systematic review and meta-analysis

**DOI:** 10.1097/MD.0000000000026410

**Published:** 2021-07-02

**Authors:** Ying-Huai Wang, Hong-Zheng Cheng, Kun Liu, Bin-Lin Cai, Yi Luo, Dan Kan, Jie Zhang

**Affiliations:** Department of Otolaryngology, Wuhan Puren Hospital, Wuhan 430081, Hubei, China.

**Keywords:** acupuncture, dysphagia, meta, nasopharyngeal carcinoma, radiotherapy

## Abstract

**Background:**

Dysphagia is a commonly occurring condition in nasopharyngeal carcinoma (NPC) patients after radiotherapy. There has been an increasing number of studies focused on assessing the use of acupuncture to manage dysphagia. Moreover, the quality of the research has gradually increased. The present research will be conducted to systematically evaluate the efficiency and safety of using acupuncture to treat cases of dysphagia after radiation therapy in NPC patients.

**Methods:**

Literature search will include all potential randomized controlled trials using MEDLINE, Cochrane Library, Web of Science, EMBASE, Chinese National Knowledge Infrastructure, Chinese BioMedical Literature, and WanFang database from their inception to May, 2021 without language or publication status restrictions, to evaluate the efficiency and safety of using acupuncture to treat dysphagia cases following radiation therapy in NPC patients. A couple of independent authors will select related literature, extract data from studies, and estimate this risk in the bias of the selected study articles. In the instance of contrasting opinions, the outcome is mediated through discussion with a different independent author. The data synthesis and statistical analysis will be completed with the RevMan software (version 5.3).

**Results:**

This study will evaluate the efficiency and safety of using acupuncture to treat dysphagia cases after radiation therapy in NPC patients.

**Conclusion:**

This study will determine the suitability of acupuncture as an effective and safe intervention for dysphagia in NPC patients after radiotherapy.

**Ethics and dissemination:**

The present study does not need ethical approval.

**Registration number:**

May 19, 2021.osf.io/f2cvt. (https://osf.io/f2cvt/).

## 1 Introduction

Nasopharyngeal carcinoma (NPC) refers to a malevolent carcinoma that arises from the nasopharyngeal epithelial layer of the nasopharynx with a distinctive topographical spreading and ethnic incidence. As shown by the IARC and GLOBOCAN cancer estimates, nearly 129,079 freshly diagnosed cases of NPC and 72,987 fatalities were reported in 2018, which was around 0.7% of total cancer diagnoses and fatalities in 2018.^[1,2]^ NPC is a type of tumor that shows high sensitivity to radiation therapy and chemotherapy. Radiation therapy, either singular or collective use of radio- and chemo-therapies are the primary methods of treatment for locally advanced NPC patients in the initial stage.^[3,4]^ Presently, intensity-modulated radiotherapy is widely adopted by most practitioners. Intensity-modulated radiotherapy has diminished the 5-year incidence rates of locoregional failure for freshly diagnosed and non-metastatic NPC to approximately 7.0%.^[5]^ The widely encountered radiotherapy-associated complications in radiotherapy for NPC consist of oral ulcers, difficulty in opening the mouth, and neck muscle fibrosis, these conditions have seriously affected the patients’ quality of life.^[6,7]^ Among them, dysphagia is considered to be the most serious complication. Moreover, a series of follow-up reactions will affect the continuity of patient treatment and effectiveness, and even be life-threatening.^[8,9]^

Acupuncture is one of the non-pharmacologic therapeutic modalities. It is primarily based on the use of fine needles on acupoints. Acupuncture at the acupoints can trigger nerve fibers and peripheral afferent receptors, stimulate sensory engagement on different levels in the central nervous system, and trigger numerous transmitters, which produces anti-inflammatory, neuro-endocrine, and neuro-immune signals.^[10]^ There is clinical evidence to prove that acupuncture reduces the side effects of cancer therapy, including pain, nausea, xerostomia, fatigue, anxiety, depression disorders, and dysphagia.^[11,12]^ However, there are varying results from studies investigating the medical healing effects of acupuncture in the treatment of dysphagia patients after radiation therapy in NPC. This can be caused by the variety of acupuncture methods used and the selection of points for acupuncture therapy. Therefore, we plan to conduct the present study to evaluate the beneficial medical effects of using acupuncture to treat dysphagia following radiation therapy in NPC patients.

## 2 Methods

### 2.1 Study registration

The current study is registered under the Open Science Framework (OSF, https://osf.io) as 10.17605/OSF.IO/F2CVT. This study adheres to the Preferred Reporting Items for Systematic Review and Meta-Analyses guidelines.^[13]^

### 2.2 Inclusion criteria for study selection

#### 2.2.1 Types of participants

This study will include all patients conformed to explicit histological-diagnosis criteria of NPC and clinical-diagnosis criteria of dysphagia. Dysphagia was diagnosed using a fiberoptic endoscopic examination of swallowing, a video-fluoroscopic swallowing study, or a clinical bedside swallowing assessment.

#### 2.2.2 Types of Interventions

The empirical collection included patients treated solely with acupuncture or in combination with different medications and therapy. The control group will be treated without treatment, placebo, or other interventions.

#### 2.2.3 Types of outcome measures

The major outcomes include MD Anderson Dysphagia Inventory, Watian swallowing test, standardized swallowing assessment, and penetration-aspiration scale. The minor outcomes include standard of life, symptoms, and adversative outcomes.

#### 2.2.4 Types of studies

The present protocol study will consist of all the randomized controlled trials (RCTs) evaluating the efficacy of using acupuncture for dysphagia after radiation therapy in NPC and exclude non-randomized studies, animal studies, observational studies, and letters.

### 2.3 Search methods for identification of studies

#### 2.3.1 Electronic searches

The literature search will include all potential RCTs using MEDLINE, Cochrane Library, Web of Science, EMBASE, Chinese National Knowledge Infrastructure, Chinese BioMedical Literature, and WanFang database from their inception to May, 2021 without language or publication status restrictions, to evaluate the efficiency and safety of using acupuncture to treat dysphagia cases following radiation therapy in NPC patients. The keywords used to search the databases are as follows: (“dysphagia” OR “swallowing disorders” OR “swallowing dysfunction”) AND “nasopharyngeal carcinoma” AND (“randomized clinical trial” OR “randomized controlled trial” OR “randomized” OR “RCT”).

#### 2.3.2 Searching other resources

The bibliography of the selected studies and related systematic reviews are collected and assessed to seek additional trials. Moreover, a search will be conducted on ClinicalTrials.gov (www.ClinicalTrials.gov) to find ongoing studies related to the topic.

### 2.4 Data collection and analysis

#### 2.4.1 Selection of studies

**Figure 1:**
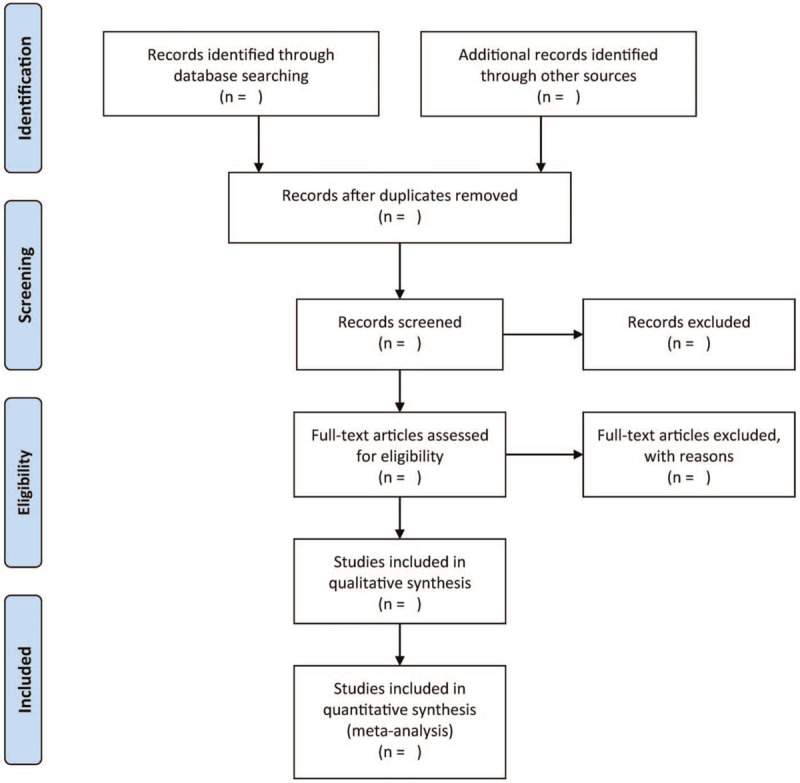
Flow diagram of study selection.

A pair of independent authors will screen the study titles, abstracts, and keywords in each of the retrieved records based on the previously designed eligibility criterion. Studies that satisfy the inclusion conditions will be selected for an additional examination of the complete text. In the case of different opinions, we will mediate the differences through mutual discussion or by consulting with another author. Figure 1 shows the process of screening studies in a flow diagram.

#### 2.4.2 Data extraction and management

A pair of independent authors will perform data extraction from the selected researches and input information into the data collection form: overall info (initial author, date published, origin, ethnicity, and country), participants (size of the sample, mean age, gender, eligibility criterion, illegibility criterion, and baseline status), methods (design, duration, and setting), intercessions (acupuncture types, names and number of acupuncture points, and treatment duration and frequency), and outcomes (events, major outcomes, adversities, and follow-up). In the case of different opinions, mediation will be reached between the 2 authors through mutual discussions or by consulting another author.

#### 2.4.3 Assessment of risk of bias in included studies

This study will utilize the criteria outlined in the Cochrane Collaboration's tool to complete a risk assessment of bias in all the studies. If there is any disagreement, all disagreements will be resolved through discussion between the pair of authors or by consulting with another author.

#### 2.4.4 Measures of treatment effect

We plan to represent dichotomous outcomes in the form of relative risk with 95% confidence intervals (CIs). Meanwhile, for continuous outcomes, the results will be presented as mean differences (MDs) or standardized MDs with 95% CIs.

#### 2.4.5 Dealing with missing data

In the case of missing data, attempts will be made to get in touch with the respective authors to collect information through electronic mail. In the event when missing data is not present, the analysis will depend on the accessible data.

#### 2.4.6 Assessment of heterogeneity

We will plan to assess heterogeneity using the Chi-squared statistic and *I*^2^ statistic that is included in the forest plot. Accordingly, a level of heterogeneity above 50% will be regarded as substantial, and then the random-effects model will be utilized for examination; else, the fixed-effects model will be utilized.^[14,15]^

#### 2.4.7 Assessment of reporting bias

Funnel plots shall be utilized to examine the potential publication bias when there are more than ten studies included in the analysis.

#### 2.4.8 Sensitivity analysis

This protocol will also perform a sensitivity analysis to determine the sensitivity of the outcomes in the event of insufficient data.

## 3 Discussion

Admittedly, previous research has provided insights into the efficiency and safety of using acupuncture to treat dysphagia cases after radiation therapy in NPC patients. However, there is controversy surrounding the results. Besides, there has been no systematic review to systematically assess the clinical therapeutic effects of using acupuncture to treat dysphagia cases following radiation therapy in NPC patients. Accordingly, the present meta-analysis will assess the efficiency and safeness associated with using acupuncture to treat dysphagia cases following radiation therapy in NPC patients. The outcomes of the current study could provide indication for clinicians and medical practitioners when taking clinical decisions to enhance the treatment of dysphagia patients.

## Author contributions

**Conceptualization:** Ying-Huai Wang, Jie Zhang.

**Data curation:** Ying-Huai Wang, Dan Kan, Jie Zhang.

**Formal analysis:** Ying-Huai Wang, Hong-Zheng Cheng, Kun Liu.

**Funding acquisition:** Kun Liu, Bin-Lin Cai, Yi Luo.

**Investigation:** Dan Kan.

**Methodology:** Hong-Zheng Cheng, Jie Zhang.

**Project administration:** Dan Kan.

**Resources:** Kun Liu, Yi Luo, Dan Kan.

**Software:** Ying-Huai Wang, Kun Liu.

**Supervision:** Hong-Zheng Cheng, Dan Kan.

**Validation:** Ying-Huai Wang, Hong-Zheng Cheng, Bin-Lin Cai, Dan Kan, Jie Zhang.

**Visualization:** Kun Liu, Yi Luo, Dan Kan.

**Writing – original draft:** Ying-Huai Wang, Bin-Lin Cai.

**Writing – review & editing:** Jie Zhang.
